# Radiomics insight into the neurodegenerative “*hot”* brain: A narrative review from the nuclear medicine perspective

**DOI:** 10.3389/fnume.2023.1143256

**Published:** 2023-02-27

**Authors:** Gayane Aghakhanyan, Gianfranco Di Salle, Salvatore Claudio Fanni, Roberto Francischello, Dania Cioni, Mirco Cosottini, Duccio Volterrani, Emanuele Neri

**Affiliations:** ^1^ Academic Radiology, Department of Translational Research and of New Surgical and Medical Technology, University of Pisa, Pisa, Italy; ^2^ Neuroradiology Unit, Department of Translational Research and New Technologies in Medicine and Surgery, University of Pisa, Pisa, Italy; ^3^ Regional Center of Nuclear Medicine, Department of Translational Research and New Technologies in Medicine and Surgery, University of Pisa, Pisa, Italy

**Keywords:** radiomics, nuclear medicine, neurodegenerative disease, Alzheimer’s disease (AD), Parkinson’s disease (PD)

## Abstract

The application of radiomics for non-oncologic diseases is currently emerging. Despite its relative infancy state, the evidence highlights the potential of radiomics approaches to serve as neuroimaging biomarkers in the field of the neurodegenerative brain. This systematic review presents the last progress and potential application of radiomics in the field of neurodegenerative nuclear imaging applied to positron-emission tomography (PET) and single-photon emission computed tomography (SPECT) by focusing mainly on the two most common neurodegenerative disorders, Alzheimer's (AD) and Parkinson's disease (PD). A comprehensive review of the current literature was performed using the PubMed and Web of Science databases up to November 2022. The final collection of eighteen relevant publications was grouped as AD-related and PD-related. The main efforts in the field of AD dealt with radiomics-based early diagnosis of preclinical AD and the prediction of MCI to AD conversion, meanwhile, in the setting of PD, the radiomics techniques have been used in the attempt to improve the assessment of PD diagnosis, the differential diagnosis between PD and other parkinsonism, severity assessment, and outcome prediction. Although limited evidence with relatively small cohort studies, it seems that radiomics-based analysis using nuclear medicine tools, mainly [18F]Fluorodeoxyglucose (FDG) and *β*-amyloid (A*β*) PET, and dopamine transporter (DAT) SPECT, can be used for computer-aided diagnoses in AD-continuum and parkinsonian disorders. Combining nuclear radiomics analysis with clinical factors and introducing a multimodality approach can significantly improve classification and prediction efficiency in neurodegenerative disorders.

## 1. Introduction

In the past decade, the field of medical image analysis using radiomics has grown exponentially enabling the conversion of digital medical images into mineable high-dimensional data (1). Radiomics is a quantitative approach to medical imaging and aims to enhance the contained information otherwise non-appreciable by the naked eye through advanced mathematical analysis (2). Although radiomics can be applied to many conditions, it is most well-developed in oncological imaging offering an infinite supply of imaging biomarkers that could potentially aid cancer detection, diagnosis, prognosis, response prediction, and monitoring of disease (1). The imaging biomarkers extracted from radiomics analysis may reflect the genotype and phenotype heterogeneity, and microenvironment, expressed, at the biological level, by cellular density, proliferation, angiogenesis, hypoxia, receptor expression, necrosis, fibrosis, and inflammation (3). Radiomics has achieved significant momentum in oncological imaging research, meanwhile, the application for non-oncologic diseases is currently emerging. Since the capability of radiomics to use high-dimensional data mining of radiological features prone to represent aging progression and contain unique information about spatial change rate at the microscopic level (4, 5) its endorsement for other multi-factorial diseases, such as neurodegenerative disorders, seems to be a natural evolution (6).

Radiomics applied to neurodegenerative brain imaging is in its relative infancy state with most of the studies focused on MRI-based features for modeling classification and/or prediction algorithms in the areas of Alzheimer's (AD) and Parkinson's disease (PD) – the two most common neurodegenerative disorders (6). AD is the most common cause of dementia, which results in memory loss, cognitive impairment, and behavioral changes. The population of patients with dementia is estimated to exceed 50 million worldwide and is expected to increase to 152 million by 2050 (7). PD is the second most common degenerative neurological disorder after AD, which is characterized by progressive motor symptoms over time. It is estimated that PD affects 1% of the population over the age of 60 with an estimated prevalence ranging from 7 to 10 million people worldwide. Pronounced increasing trends of PD burden are observed worldwide, and in most regions and countries, indicating that PD is an increasing challenge to global health (8).

Converging evidence highlights the potential of radiomics approaches to serve as neuroimaging biomarkers in the field of the neurodegenerative brain (9–12). This narrative review presents the last progress and potential application of radiomics in the field of neurodegenerative nuclear imaging focusing mainly on positron-emission tomography (PET) and single-photon emission computerized tomography (SPECT) with different radiotracers. As the central point of this narrative review was radiomics with the explicit use of radiomics feature extraction, we did not cover other AI-based approaches, such as deep neuronal network (DNN), which represent the next-door evolving field in medical imaging analysis for the neurodegenerative brain.

## 2. Material and methods

**Figure 1:**
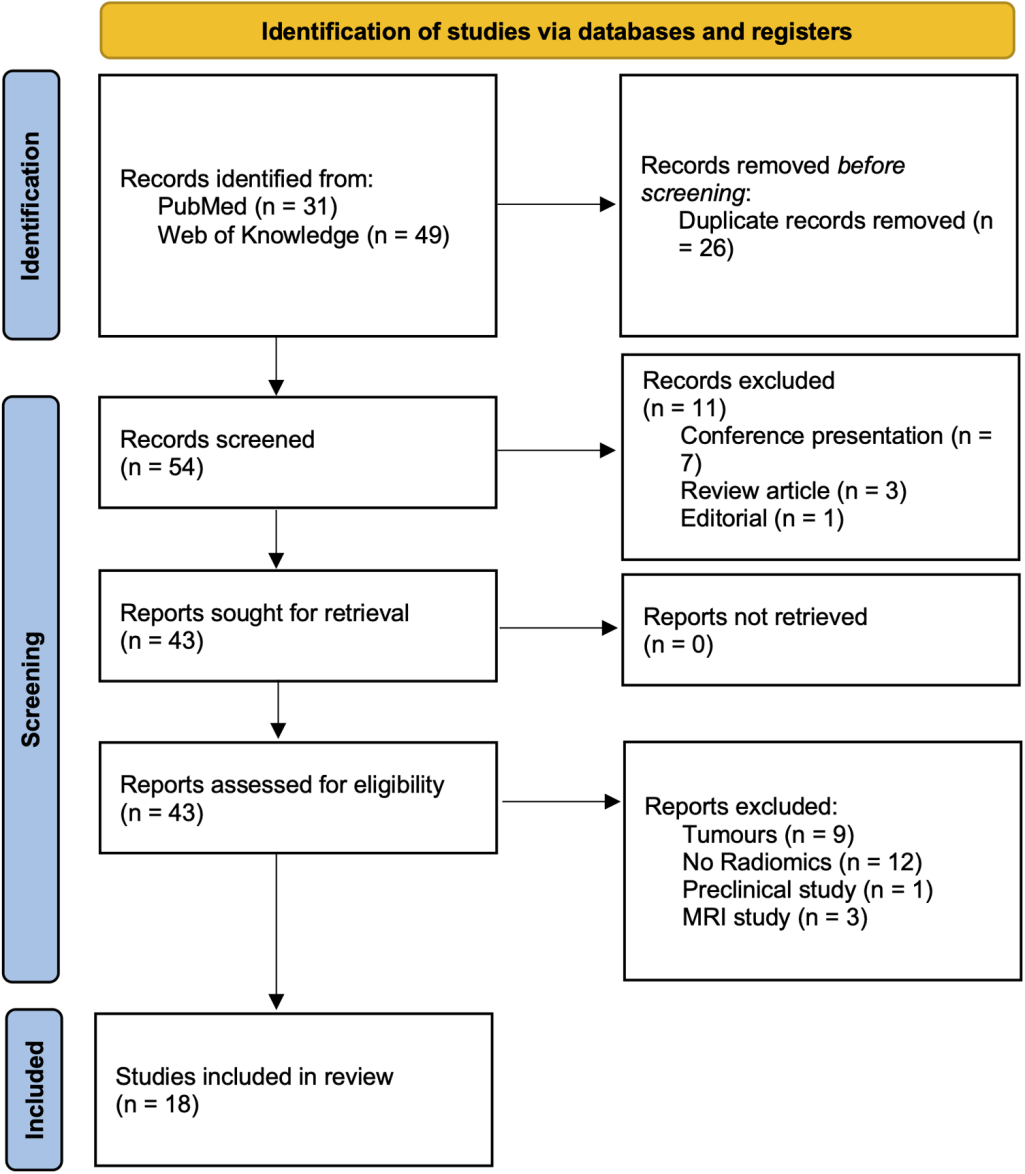
PRISMA flow diagram.

A comprehensive review of the current literature was performed using the PubMed and Web of Science databases up to November 2022 using the following search criteria: (radiomic* OR radiogenomic*) AND (“Neurodegenerative Diseases” [Mesh] OR “Dementia” [Mesh] OR Alzheimer's* OR “Basal Ganglia Diseases” [Mesh] OR Parkinson*) AND [“Tomography, Emission-Computed” (Mesh) OR PET OR DAT]. The exclusion criteria were as follows: unavailability of full text; non-English publications; image processing was not covering image segmentation and radiomics feature extraction; publications unrelated to the field of neurodegenerative disorders; imaging modalities other than nuclear medicine imaging tools; reviews, conference abstracts, and editorials. Following the PRISMA criteria (13), Figure 1 was included to delineate the article selection process (Figure 1).

## 3. Narrative synthesis of the results

The final collection of 18 relevant publications were included and summarized in Table 1. Overall, *n* = 54 titles and abstracts were screened, and *n* = 43 full papers were assessed. The records including tumor (*n* = 9), non-radiomics study (*n* = 12), preclinical study (*n* = 1), and MRI protocol (*n* = 3) were excluded. The first study started in 2016, reflecting the fact that radiomics is a relatively new approach in the field of nuclear neurology. Selected papers were grouped as AD-related (*n* = 9) and PD-related (*n* = 9).

**Table 1:** Study characteristics.

First author	Year	Title	Group	Number of cases	Tool	Task	Source
Alongi et al.	2022	Radiomics Analysis of Brain [ (18)F]FDG PET/CT to Predict Alzheimer's Disease in Patients with Amyloid PET Positivity: A Preliminary Report on the Application of SPM Cortical Segmentation, Pyradiomics and Machine-Learning Analysis.	AD-related	43	[18F]FDG PET	Prediction	local
Ciarmiello et al.	2022	Machine Learning Model to Predict Diagnosis of Mild Cognitive Impairment by Using Radiomic and Amyloid Brain PET.	AD-related	328	[18]F Florbetaben (FBB) PET	Classification	ADNI^a^
Jiang et al.	2022	Using radiomics-based modelling to predict individual progression from mild cognitive impairment to Alzheimer's disease.	AD-related	884	[18F]FDG PET	Classification and Prediction	ADNI
Sheng et al	2022	Cross-Cultural Longitudinal Study on Cognitive Decline (CLoCODE) for Subjective Cognitive Decline in China and Germany: A Protocol for Study Design.	AD-related	479	[18]F-AV-45 (florbetapir) PET	Classification and Correlation	ADNI
Yang et al.	2022	Combining PET with MRI to improve predictions of progression from mild cognitive impairment to Alzheimer's disease: an exploratory radiomic analysis study.	AD-related	471	[18F]FDG PET	Prediction and Correlation	local, ADNI
Ding et al.	2021	Quantitative Radiomic Features as New Biomarkers for Alzheimer's Disease: An Amyloid PET Study.	AD-related	1078	[18]F-AV-45 (florbetapir) PET	Classification	local, ADNI
Huang et al	2021	Radiogenomics of Alzheimer's disease: exploring gene related metabolic imaging markers.	AD-related	389	[18F]FDG PET	Prediction	multicenter
Li et al.	2019	Radiomics: a novel feature extraction method for brain neuron degeneration disease using (18)F-FDG PET imaging and its implementation for Alzheimer's disease and mild cognitive impairment.	AD-related	466	[18F]FDG PET	Prediction	local, ADNI
Zhou et al.	2019	Dual-Model Radiomic Biomarkers Predict Development of Mild Cognitive Impairment Progression to Alzheimer's Disease.	AD-related	263	[18F]FDG PET	Prediction	ADNI
Comte et al.	2022	Development and validation of a radiomic model for the diagnosis of dopaminergic denervation on [18F]FDOPA PET/CT.	PD-related	443	[18F]FDOPA PET	Classification	local
Salmanpour et al.	2022	Longitudinal clustering analysis and prediction of Parkinson's disease progression using radiomics and hybrid machine learning.	PD-related	143	DAT-SPECT (123I-Ioflupane)	Classification	local
Shiiba et al.	2022	Dopamine transporter single-photon emission computed tomography-derived radiomics signature for detecting Parkinson's disease.	PD-related	413	DAT-SPECT (123I-Ioflupane)	Prediction	PPMI^b^
Hu et al.	2021	Multivariate radiomics models based on (18)F-FDG hybrid PET/MRI for distinguishing between Parkinson's disease and multiple system atrophy.	PD-related	90	[18F]FDG PET/CT	Prediction and Correlation	local
Salmanpour et al.	2021	Robust identification of Parkinson's disease subtypes using radiomics and hybrid machine learning.	PD-related	464	DAT-SPECT (123I-Ioflupane)	Clustering	PPMI
Tang et al.	2019	Artificial Neural Network-Based Prediction of Outcome in Parkinson's Disease Patients Using DaTscan SPECT Imaging Features.	PD-related	69	DAT-SPECT (123I-Ioflupane)	Clustering and Prediction	PPMI
Wu et al.	2019	Use of radiomic features and support vector machine to distinguish Parkinson's disease cases from normal controls.	PD-related	230	[18F]FDG PET/CT	Classification	local
Rahmim et al.	2017	Improved prediction of outcome in Parkinson's disease using radiomics analysis of longitudinal DAT SPECT images.	PD-related	64	DAT-SPECT (123I-Ioflupane)	Prediction	PPMI
Rahmim et al.	2016	Application of texture analysis to DAT SPECT imaging: Relationship to clinical assessments	PD-related	141	DAT-SPECT (123I-Ioflupane)	Classification	multicenter

^a^ The Alzheimer's Disease Neuroimaging Initiative (ADNI) database, www.adni.loni.usc.edu.

^b^ The Parkinson's Progression Markers Initiative (PPMI) database, www.ppmi-info.org.

## 4. Discussion

### 4.1. Radiomics in Alzheimer's disease

The application of radiomics to analyze the complex patterns of PET imaging of the AD brain is in its early stages. So far, few studies have investigated the potential of radiomics using [18F]Fluorodeoxyglucose (FDG) PET, and to a lesser extent, *β*-amyloid (A*β*) PET brain images for the evaluation of neurodegenerative diseases. At the time of selecting papers for the current narrative review, there was overall a lack of studies that applied radiomics for AD spectrum disorders beyond the FDG and A*β* PET, despite the inherent potential of radiomics to unveil quantitative imaging biomarkers, if any, using different modalities and different compounds.

In recent years, the exchange of scientific views was mainly focused on radiomics applied in early AD diagnosis and prediction of mild cognitive impairment (MCI) conversion. Early in-vivo diagnosis of AD is critical for accurate patient management and the advanced radiomic features could fully account for brain tissue heterogeneity allowing for the identification of MCI patients who are likely to convert to AD. Despite the simplicity of the acquisition of radiomic features, the high-throughput nature, relative stability of the radiomics-based classification and prediction algorithms, their role in precision medicine for the population affected by AD yet remains at an exploratory stage of development (14).

#### 4.1.1. Radiomics-based classification for AD spectrum

Automated classification of AD, nonetheless, in the evolving state, has shown promise with AI algorithms to accurately differentiate between individuals who have been clinically diagnosed with AD and MCI from those of healthy controls. Currently, more extensive work for approaching the classification tasks was done using DNN that can automatically learn discriminatory multilevel and multimodal features with high accuracy (15, 16). The advantage of DNN algorithms is that these models do not require segmentation and are robust to scale and rotation variations (15). However, the “black-box” nature of DNNs makes them less trustworthy to physicians, thus hindering their expansion into real clinical settings. On the other side, the attractiveness of radiomics-based algorithms is that they can serve as a potential bridge between brain imaging and personalized medicine, as these algorithms are able to incorporate patient-related information and disease-related biomarkers.

The mainstream pipelines using radiomics, first, segment the PET brain image into various regions of interest (ROIs), extract and select the discriminate features for AD-target ROI regions, and finally, input to state-of-the-art machine learning models trained to support different tasks for clinical decision-making (17). A few studies using ROI-based radiomics and support vector machine (SVM) classifiers with selected radiomic features extracted from [18F]FDG PET images reported stability achieving good classification accuracies for classifying AD vs. HC (91.5 to 92.9%), MCI vs. HC (83.1 to 83.7%), AD vs. MCI (85.9 to 87.9%), and MCI_converter_ vs. MCI_non−converter_ (88%) (18, 19). Similarly, Ciarmiello and co-authors demonstrated that ROI-based radiomics using gray-level run-length matrix for texture analysis and feed-forward multilayer neural network on A*β*-PET data outperform SUVr-based performance (AUC 0.9 vs. 0.71) in differentiation amnestic MCI subjects from healthy controls (20).

Besides conventional ROI-based radiomics, Ding et al. applied voxel-wise radiomics on standard uptake value ratio (SUVr) intensity data using A*β*-PET (9) as a biomarker to classify healthy controls and AD with a nonlinear SVM model and cross-validation techniques. The classification performance was better than that with only the A*β* scores based on regions (ACC = 0.75, SPE = 0.80, SEN = 0.70, AUC = 0.81) or based on voxels (ACC = 0.84, SPE = 0.86, SEN = 0.81, AUC = 0.90).

#### 4.1.2. Radiomics-based prediction of MCI to AD conversion

It is a significant challenge to evaluate and predict the progression of MCI for early treatment of AD (9, 19). A classic approach for predicting MCI conversion is to train a classification model using the data of MCI converters and non-converters and then use the trained classifier to classify new MCI patients (Liu et al. 2018). In addition, the proportional hazards (Cox) model is commonly constructed as the prediction model for investigating the effect of several variables (predictive features) upon the time (conversion time) (21).

Jiang and co-authors designed different Cox models (radiomics, clinical, SUVr FDG PET) to compare the predictive performance of MCI conversion (21). The experimental results showed that the predictive performance of the radiomics-based Cox model was better than that of other Cox models. In the validation dataset, Harrell's consistency coefficient of the radiomics-based Cox model was 0.703 ± 0.002, while those of the clinical and SUVR models were 0.632 ± 0.006 and 0.683 ± 0.009, respectively.

Alongi et al. propose a radiomics approach on [18F]FDG PET/CT brain images based on Statistical Parametric Mapping (SPM) and Pyradiomics to predict PET-Amyloid positivity (22). The authors found six radiomics features that predict cortical A*β* deposition with high sensitivity, specificity, precision, and accuracy of 84.92%, 75.13%, 73.75%, and 79.56%, respectively. These preliminary results obtained from [18F]FDG PET/CT brain radiomics for predicting the presence of A*β* deposition may serve as a new approach for selecting those patients who can benefit from the diagnostic use of A*β* PET.

The workflow for radiomics-based predictive analysis using A*β* PET brain images was also reported (9). The A*β* PET radiomic features with widely used machine learning and cross-validation techniques are able to predict with good performance (AUC 0.83) the progression of high-risk MCI patients' conversion to AD (9). Interestingly, in the MCI and AD groups, the classification outputs are significantly associated with clinical measures, such as apolipoprotein E genotype, polygenic risk scores, polygenic hazard scores, cerebrospinal fluid A*β*, and Tau, cognitive ability score, the conversion time for progressive MCI subjects and cognitive changes, thus, highlighting the solid biological/clinical basis underlying the progression of AD (9).

A few studies attempt to address the added value of dual-modality imaging for the prediction of MCI conversion to AD by using radiomic analysis (14, 23). Yang and co-authors compared the predictive accuracy of single-modality MRI and [18F]FDG PET and dual-modality [18F]FDG PET/MRI to predict the MCI conversion to AD, demonstrating a large overlap between [18F]FDG PET and MRI radiomics model performance, while the dual-modality model resulted in only a modest improvement over the single-modality models (Harrell's C-index of 0.798 for dual-modality vs. 0.760 for MRI, and 0.734 for PET; both *P* < 0.001) (14). Even though the incremental benefit of combining [18F]FDG PET and MRI for predicting MCI conversion seems limited according to Yang et. al., the integration of cross-modality approaches using radiomics should be prioritized for their potential additive value allowing the combination of the extracted imaging information for each modality. Furthermore, as radiomics is naturally designed to develop decision-support tools for precision medicine, it should involve the combination of radiomic data with other patient characteristics, as available, to increase the power of the decision-support models (14). Zhou and co-authors showed improved performance constructed similar prognostic Cox models, yet, using the clinical data, MRI images, PET images, fused MRI/PET images, and clinical variables and fused MRI/PET images in combination (Harrell's C-index 0.69, 0.73, 0.73 and 0.75, and 0.78, respectively) (23). Significant enhancement in the prediction of conversion of the imaging models (MRI/PET/fused) compared to clinical models was observed, and the combination of fused-modality imaging and clinical variables resulted in the greatest accuracy of prediction. According to the authors, the combination of radiomic and Cox model analyses could be used successfully in survival analysis and may be a powerful tool for personalized precision medicine patients with the potential to undergo conversion from MCI to AD (23).

### 4.2. Radiomics in Parkinson's disease and atypical parkinsonisms

In the last decade, radiomics techniques have been used in the attempt to improve the assessment of key features of PD patients, namely: PD diagnosis, the differential diagnosis between PD and other parkinsonisms, severity assessment, and outcome prediction. In this review, we summarize these attempts and evaluate their potential and actual impact on PD patients' management.

#### 4.2.1. Clinical-Imaging correlation and disease severity assessment

As the most urgent in clinical practice, the first application of radiomics in nuclear medicine imaging of PD regarded the possibility of effectively correlating imaging and clinical data after diagnosis and initial clinical assessment.

Rahmim et al. analyzed cross-sectional data from Parkinson's Progressive Marker Initiative (PPMI) and found that striatal dopamine transporter (DAT) single-photon emission computed tomography (SPECT) textural features significantly correlated with the cognitive and motor status of 85 PD patients (24). Cognitive and motor performance were measured through UPRDS (part III – motor) and Montreal Cognitive Assessment (MoCA) scores, respectively. In this work, the most effective textural metrics for clinical correlation were extracted from the caudate by the most affected side. The authors discussed their possible use for the early diagnosis of PD in subjects at increased risk (e.g., mutation carriers or RBD patients).

Salmanpour et al. used cross-sectional and timeless data to identify 3 distinct PD subtypes, defined as mild, intermediate, and severe. In this research, non-imaging, conventional imaging data and radiomics features were combined. Importantly, disease clustering was robust to variations in features and sample size (25). The inclusion of radiomics features (especially structural features derived from MRI) was essential for cluster robustness.

#### 4.2.2. Outcome prediction

Prompted by the previously cited paper from the same group, a longitudinal investigation by Rahmim et al. found that radiomics features from year-0 and year-1 DAT-SPECT images, together with clinical data and conventional imaging features, contributed to predicting the motor outcome of PD patients (26). In this model, clinical variables (year-0 and year-1 UPDRS-III, the unified Parkinson's disease rating scale) were the most predictive, but the addition of conventional imaging and radiomics features allowed to reduce of the average absolute prediction error of the year-4 UPDRS-III from 8 to 3 points.

The same attempt was made two years later, by using only year-0 clinical and imaging data but using an artificial neural network (ANN) to build a predictive model based on the radiomics features extracted from basal ganglia DAT SPECT (27). Importantly, this study was performed on data extracted from the same database (PPMI) as Rahmim et al. (26). The ANN reached an accuracy of 75% in predicting year-4 UPDRS-III class (<30 vs. ≥30), with a clear benefit from the combination of imaging and non-imaging (year-0 UPDRS-III) features to the model.

In recent work, Salmanpour et al. applied their clustering approach to the outcome prediction problem, and divided PD patients from PPMI database into 3 distinct progression trajectories over a 4-years follow-up, based on the motor, and non-motor clinical features, and radiomics features (28). The authors also used clinical and imaging features to build a model for the prediction of the disease trajectory, reaching an accuracy of 79%. In trajectory I (35% of patients), the disease was stable for the whole follow-up duration; in trajectory II (27%), patients underwent a clinical-imaging improvement for the first 2 years and then worsened to severe disease at 4 years; in trajectory 3 (38%), patients showed a monophasic worsening throughout the follow-up. Importantly, the consistency of the three cross-sectional disease subtypes described in previous work was observed at each timepoint in the follow-up (25). While these clusters remained distinct, longitudinal trajectories allowed patients to move across PD subtypes through time. Considering this finding, timeless disease subtyping can be seen as a multidimensional phenotypic description, which is not necessarily correlated to longitudinal progression.

#### 4.2.3. Diagnosis and early differentiation from atypical Parkinsonisms

[18F]FDG-PET radiomics was first tested for the diagnosis of PD vs. HC by Wu et al. (29). They found that, in multiple ROIs, features like *LGZE* (low gray-level zone emphasis), *skewness* (a measure of asymmetry) and *LRHGE* (long-run high gray-level emphasis) were predictive of PD diagnosis with an overall accuracy of 88%. Notably, some of these features were also significantly correlated with UPDRS (part III) and uptake values in the ROIs. Recently, Comte et al. used textural features from FDOPA PET to predict dopaminergic denervation (30). Conventional imaging features performed poorly, but the textural features allowed the model to reach an accuracy of 95.83%. Notably, no model was built here for subtyping the dopaminergic denervation into PD/ atypical-parkinsonisms. Shiiba et al. compared DAT-SPECT conventional features and a radiomics signature for the classification of 413 PPMI patients into PD or HC (31). Radiomics signature included features mostly from putamen and pallidum and was up to 96.8% accurate in the discrimination of PD patients. Nevertheless, it failed to outperform the conventional striatal uptake ratio (SUR).

Although automated image-based classification methods have been investigated for more than a decade in the differential diagnosis of PD and atypical parkinsonism, the first radiomics-based approach to this problem was by Hu et al. (32). Here, a combined clinical and [18F]FDG PET/MRI model with imaging features from PET, T1- and susceptibility-weighted imaging reached an accuracy of 96.3% in the differential diagnosis between PD and multiple system atrophy (MSA).

In a study with DAT PET, Zhao et al., found differences in basal ganglia relative binding ratios among patients with different parkinsonisms but failed to demonstrate differences between PD and MSA (33). Despite the authors extracting conventional radiomics features which were significantly different among atypical parkinsonisms no predictive model was built for the differential diagnosis, because the study was centered on deep learning-based applications.

## 5. Comparative model performance

The model comparison is commonly used in radiomics studies to compare the explanatory power of two or more models, with the goal of identifying the model that best explains the variance. Comparison of the classification and prediction performance usually achieved by constructing different single- and/or multiple modality models including clinical, conventional imaging, radiomics and other available parameters. It has been shown that radiomics-based models regularly overperform conventional SUVr-based regional models (20, 21, 31), as well as single clinical models that usually incorporate age, gender, education, Mini-Mental State Examination (MMSE), MoCA, UPDRS (for PD) and APOE *ε*4 genotype (14, 18, 23, 24, 27). The growing evidence suggests that the best performance is achieved with combined models using clinical, conventional imaging (MRI-based, DAT-SPECT and SUVr PET-based models) and radiomics parameters in comparison to single-modality models (14, 18, 23), which is foreseeable, as a model with more parameters will almost always explain slightly more variance than a model with less parameters.

## 6. Conclusions

Despite limited evidence with relatively small cohort studies, it seems that radiomics-based analysis using nuclear medicine tools can be used for computer-aided diagnoses in AD-continuum and parkinsonian disorders. Combining radiomics analysis with clinical factors and introducing a multimodality approach can significantly improve classification efficiency leading to potential applications of radiomics-based early diagnosis of preclinical AD, prediction of MCI to AD conversion, assessing disease severity and early differentiation of different parkinsonian disorders.
